# Single-dose AAV-based vaccine induces a high level of neutralizing antibodies against SARS-CoV-2 in rhesus macaques

**DOI:** 10.1093/procel/pwac020

**Published:** 2022-07-15

**Authors:** Dali Tong, Mei Zhang, Yunru Yang, Han Xia, Haiyang Tong, Huajun Zhang, Weihong Zeng, Muziying Liu, Yan Wu, Huan Ma, Xue Hu, Weiyong Liu, Yuan Cai, Yanfeng Yao, Yichuan Yao, Kunpeng Liu, Shifang Shan, Yajuan Li, Ge Gao, Weiwei Guo, Yun Peng, Shaohong Chen, Juhong Rao, Jiaxuan Zhao, Juan Min, Qingjun Zhu, Yanmin Zheng, Lianxin Liu, Chao Shan, Kai Zhong, Zilong Qiu, Tengchuan Jin, Sandra Chiu, Zhiming Yuan, Tian Xue

**Affiliations:** First Affiliated Hospital of USTC, School of Life Sciences, Division of Life Sciences and Medicine, University of Science and Technology of China, Hefei 230026, China; First Affiliated Hospital of USTC, School of Life Sciences, Division of Life Sciences and Medicine, University of Science and Technology of China, Hefei 230026, China; Hefei National Research Center for Physical Sciences at the Microscale, Neurodegenerative Disorder Research Center, CAS Key Laboratory of Brain Function and Disease, CAS Key Laboratory of Innate Immunity and Chronic Disease, Biomedical Sciences and Health Laboratory of Anhui Province, University of Science and Technology of China, Hefei 230026, China; First Affiliated Hospital of USTC, School of Life Sciences, Division of Life Sciences and Medicine, University of Science and Technology of China, Hefei 230026, China; Hefei National Research Center for Physical Sciences at the Microscale, Neurodegenerative Disorder Research Center, CAS Key Laboratory of Brain Function and Disease, CAS Key Laboratory of Innate Immunity and Chronic Disease, Biomedical Sciences and Health Laboratory of Anhui Province, University of Science and Technology of China, Hefei 230026, China; State Key Laboratory of Virology, Wuhan Institute of Virology, Chinese Academy of Sciences, Wuhan 430071, China; Center for Biosafety Mega-Science, Wuhan Institute of Virology, Chinese Academy of Sciences, Wuhan 430071, China; Hefei Institutes of Physical Science, Chinese Academy of Sciences, Hefei 230031, China; Center for Biosafety Mega-Science, Wuhan Institute of Virology, Chinese Academy of Sciences, Wuhan 430071, China; Hefei National Research Center for Physical Sciences at the Microscale, Neurodegenerative Disorder Research Center, CAS Key Laboratory of Brain Function and Disease, CAS Key Laboratory of Innate Immunity and Chronic Disease, Biomedical Sciences and Health Laboratory of Anhui Province, University of Science and Technology of China, Hefei 230026, China; Anhui Institute of Pediatric Research, Anhui Provincial Children’s Hospital, Hefei 230051, China; Center for Biosafety Mega-Science, Wuhan Institute of Virology, Chinese Academy of Sciences, Wuhan 430071, China; Hefei National Research Center for Physical Sciences at the Microscale, Neurodegenerative Disorder Research Center, CAS Key Laboratory of Brain Function and Disease, CAS Key Laboratory of Innate Immunity and Chronic Disease, Biomedical Sciences and Health Laboratory of Anhui Province, University of Science and Technology of China, Hefei 230026, China; State Key Laboratory of Virology, Wuhan Institute of Virology, Chinese Academy of Sciences, Wuhan 430071, China; First Affiliated Hospital of USTC, School of Life Sciences, Division of Life Sciences and Medicine, University of Science and Technology of China, Hefei 230026, China; First Affiliated Hospital of USTC, School of Life Sciences, Division of Life Sciences and Medicine, University of Science and Technology of China, Hefei 230026, China; Center for Biosafety Mega-Science, Wuhan Institute of Virology, Chinese Academy of Sciences, Wuhan 430071, China; First Affiliated Hospital of USTC, School of Life Sciences, Division of Life Sciences and Medicine, University of Science and Technology of China, Hefei 230026, China; Hefei National Research Center for Physical Sciences at the Microscale, Neurodegenerative Disorder Research Center, CAS Key Laboratory of Brain Function and Disease, CAS Key Laboratory of Innate Immunity and Chronic Disease, Biomedical Sciences and Health Laboratory of Anhui Province, University of Science and Technology of China, Hefei 230026, China; State Key Laboratory of Virology, Wuhan Institute of Virology, Chinese Academy of Sciences, Wuhan 430071, China; Chinese Academy of Sciences Center for Excellence in Brain Science and Intelligence Technology, Chinese Academy of Sciences, Shanghai 200031, China; Department of Clinical Laboratory, First Affiliated Hospital of Anhui Medical University, Hefei, 230022, China; Center for Biosafety Mega-Science, Wuhan Institute of Virology, Chinese Academy of Sciences, Wuhan 430071, China; State Key Laboratory of Virology, Wuhan Institute of Virology, Chinese Academy of Sciences, Wuhan 430071, China; Center for Biosafety Mega-Science, Wuhan Institute of Virology, Chinese Academy of Sciences, Wuhan 430071, China; State Key Laboratory of Virology, Wuhan Institute of Virology, Chinese Academy of Sciences, Wuhan 430071, China; State Key Laboratory of Virology, Wuhan Institute of Virology, Chinese Academy of Sciences, Wuhan 430071, China; State Key Laboratory of Virology, Wuhan Institute of Virology, Chinese Academy of Sciences, Wuhan 430071, China; Center for Biosafety Mega-Science, Wuhan Institute of Virology, Chinese Academy of Sciences, Wuhan 430071, China; Hefei Institutes of Physical Science, Chinese Academy of Sciences, Hefei 230031, China; Hefei Institutes of Physical Science, Chinese Academy of Sciences, Hefei 230031, China; First Affiliated Hospital of USTC, School of Life Sciences, Division of Life Sciences and Medicine, University of Science and Technology of China, Hefei 230026, China; State Key Laboratory of Virology, Wuhan Institute of Virology, Chinese Academy of Sciences, Wuhan 430071, China; Hefei Institutes of Physical Science, Chinese Academy of Sciences, Hefei 230031, China; Chinese Academy of Sciences Center for Excellence in Brain Science and Intelligence Technology, Chinese Academy of Sciences, Shanghai 200031, China; First Affiliated Hospital of USTC, School of Life Sciences, Division of Life Sciences and Medicine, University of Science and Technology of China, Hefei 230026, China; Hefei National Research Center for Physical Sciences at the Microscale, Neurodegenerative Disorder Research Center, CAS Key Laboratory of Brain Function and Disease, CAS Key Laboratory of Innate Immunity and Chronic Disease, Biomedical Sciences and Health Laboratory of Anhui Province, University of Science and Technology of China, Hefei 230026, China; First Affiliated Hospital of USTC, School of Life Sciences, Division of Life Sciences and Medicine, University of Science and Technology of China, Hefei 230026, China; Hefei National Research Center for Physical Sciences at the Microscale, Neurodegenerative Disorder Research Center, CAS Key Laboratory of Brain Function and Disease, CAS Key Laboratory of Innate Immunity and Chronic Disease, Biomedical Sciences and Health Laboratory of Anhui Province, University of Science and Technology of China, Hefei 230026, China; Center for Biosafety Mega-Science, Wuhan Institute of Virology, Chinese Academy of Sciences, Wuhan 430071, China; First Affiliated Hospital of USTC, School of Life Sciences, Division of Life Sciences and Medicine, University of Science and Technology of China, Hefei 230026, China; Hefei National Research Center for Physical Sciences at the Microscale, Neurodegenerative Disorder Research Center, CAS Key Laboratory of Brain Function and Disease, CAS Key Laboratory of Innate Immunity and Chronic Disease, Biomedical Sciences and Health Laboratory of Anhui Province, University of Science and Technology of China, Hefei 230026, China; Chinese Academy of Sciences Center for Excellence in Brain Science and Intelligence Technology, Chinese Academy of Sciences, Shanghai 200031, China; Institute for Stem Cell and Regeneration, Chinese Academy of Sciences, Beijing 100101, China

**Dear Editor,**

Coronavirus disease 2019 (COVID-19) is a highly infectious respiratory disease that continues to pose a serious global public health emergency. The disease shows a high infection rate, long incubation period, and rapidly emerging variants, which have led to its rapid spread worldwide (Krammer 2020). Many vaccines have been developed for the control of severe acute respiratory syndrome coronavirus 2 (SARS-CoV-2), the virus responsible for COVID-19, including vaccines based on messenger RNA (mRNA) (Polack et al. 2020), viral vectors (Zhu et al. 2020), recombinant proteins (Yang et al. 2020), and inactivated SARS-CoV-2 (Zhang et al. 2021). Indeed, several of these vaccines have been shown to protect population from SARS-CoV-2 infection. However, most vaccines lack long-term protection efficacy (Cohn et al. 2022), and most of them require two or three injections to induce neutralizing antibodies (NAbs). Therefore, developing a vaccine that only requires a single-dose immunization and provides long-term NAbs would be optimal for combating COVID-19.

Adeno-associated virus (AAV) is a single-stranded DNA parvovirus widely used for gene therapy and vaccines. AAV ­vector-mediated gene therapy products have been approved by the Food and Drug Administration (FDA) for the treatment of inherited blindness and spinal muscular atrophy (Wang et al. 2019). AAV vectors have special features that are highly beneficial for clinical applications, such as low immunogenicity, long-lasting gene expression, safety, and high efficacy. Adenoviruses (AdVs) and AAVs are two types of viral vector used for gene delivery, with AdVs more commonly utilized for SARS-CoV-2 vaccines (Zhu et al. 2020). Both systems can infect a broad range of hosts, including dividing and non-dividing cells. However, there are several key distinctions between them, including onset and duration of gene expression, packaging capacity, and immune response. AdV vectors can accommodate larger inserts, but mediate transient protein expression and may cause severe inflammation and immune response. Compared with AdVs, AAVs exhibit longer lasting gene expression and lower immune response. Thus, we applied AAV vectors in the current study to develop a long-term expression vaccine for the prevention of COVID-19.

The SARS-CoV-2 spike protein mediates the binding of the virus to the human angiotensin converting enzyme 2 (ACE2) receptor for entry into target cells (Shang et al. 2020). As such, it is the main antigen target for vaccines. Based on the SARS-CoV-2 spike protein structure (Protein data bank [PDB]: 6VXX), we found that the receptor-binding domain (RBD) was not as stable as the domain that spanned the spike protein from Q321 to S591, with the C and N tails forming a stabilizing beta-sheet (Fig. 1A), hereafter termed SRBD. Thermal stability analysis also showed that the SRBD protein (56.88 ± 0.45°C) was more thermostable than RBD (52.28 ± 0.77°C) (Fig. 1B). The AAV2/9 serotype was chosen as the vaccine carrier due to its high transduction efficiency in muscles. To assess the immunogenicity of the designed vaccines, we injected AAV-SRBD vaccines intramuscularly into both C57BL/6J and NIH mice at a dose of 1 × 10^11^ virus genomes (vg)/mouse, respectively (Fig. S1A and S1B). AAV-CAG-GFP (1 × 10^11^ vg/mouse) was used as a control. Results showed that the AAV-SRBD could express well in the muscle of mice (Fig. 1C). Moreover, AAV-SRBD resulted in high antibody titers in both NIH and C57BL/6J mice (Fig. 1D and 1E). Thus, we used SRBD as the antigen for generating an AAV-based COVID-19 vaccine. To evaluate the tissue-specific expression patterns of AAV2/9, we analyzed the expression of AAV-CAG-GFP in several major mouse organs (Fig. S1C–E). Green fluorescent protein (GFP) signaling was found in the injected muscle cells and liver cells of mice, but not in other major organs, i.e., heart, lung, spleen, kidney, and whole brain. Moreover, histological analysis illustrated that no significant pathological changes occurred in the major tissues of AAV-injected mice, e.g., lung, heart, liver, spleen, and kidney, compared with the naïve C57BL/6J mice (Fig. S1F). These results suggest that the AAV-SRBD vaccine exhibited good immunogenicity and safety in mice.

**Figure 1.**
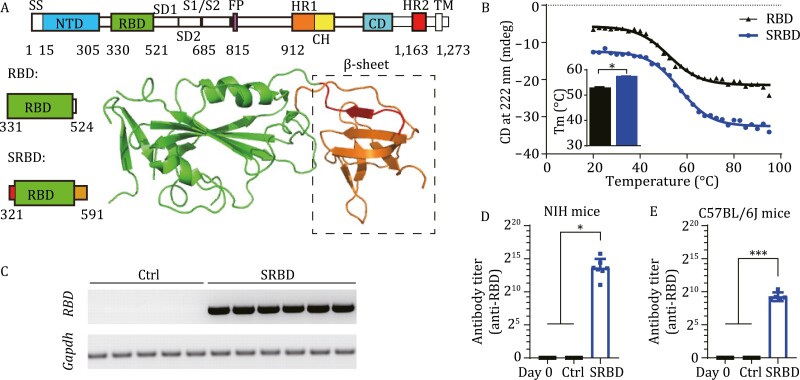
Single-dose SRBD vaccine elicits humoral responses in mice. (A) Schematic of RBD and SRBD structure. β-sheet was formed between red section (Q321 to P330) and orange section (C525 to S591), which provided stability to SRBD protein. SS, signaling sequence; NTD, N-terminal domain; RBD, receptor-binding domain; SD1, subdomain 1; SD2, subdomain 2; HR1, heptad repeat 1; CH, central helix; CD, connector domain; HR2, heptad repeat 2; TM, transmembrane domain. (B) Thermal stability analysis of RBD and SRBD proteins by circular dichroism spectroscopy. Thermal denaturation of RBD and SRBD is shown in black and blue curves, respectively, between 20 °C and 95 °C monitored at 222 nm. The thermal denaturation of RBD and SRBD were tested three times. (C) mRNA expression of RBD in muscle samples of AAV-GFP-, AAV-SRBD-injected C57BL/6J mice at 28 dpv (*n* = 5 mice in control [Ctrl] group, *n* = 6 mice in SRBD group). (D) Quantitative analysis of RBD antibody titer calculated by ELISA at 28 dpv in NIH mice (*n* = 5 mice in Ctrl group, *n* = 7 mice in SRBD group, *n* = 12 in Day 0 group). (E) Quantitative analysis of RBD antibody titer calculated by ELISA at 42 dpv in C57BL/6J mice (*n* = 5 mice in Ctrl and SRBD group, *n* = 10 in Day 0 group). Data (D to E) were obtained from one experiment. Each serum was tested three times as technical replicates. Values are means ± s.e.m. or geometric mean + geometric standard deviation for antibody titer. *P < 0.05; ***P < 0.001.

To further examine the vaccine safety and efficacy, we tested the AAV-SRBD vaccine in a nonhuman primate (NHP) species. Two groups of rhesus macaques (*Macaca mulatta*) were used for the study. The first group included three macaques with high-dose vaccine (1 × 10^12^ vg/macaque) and was used for long-term monitoring of the NAb titers. The second group included 13 macaques with different doses. Thirteen macaques were randomly divided into three groups, then received a ­single-dose immunization of 1 × 10^12^ vg/macaque (high-dose, four macaques), 1 × 10^11^ vg/macaque (middle-dose, three macaques), or 1 × 10^10^ vg/macaque (low-dose, three macaques) of AAV-SRBD, respectively. ­AAV-CAG-GFP (1 × 10^12^ vg/macaque) was used as the control (three macaques) (Table S1). The dosage dependent effect of the vaccine, the body weight, antibody titer, pathological indicators in blood and hepatic function of macaques in the second group were examined until 70 post vaccination (dpv). All ­intramuscular-injected macaques in the second group showed normal body weight post injection (Fig. S2A). Blood samples from all macaques were collected to assess the antibody titers (Fig. 2A). One potential limitation of AAV vaccine application is that most humans and macaques have experienced wild-type AAV exposure, which can result in pre-existing AAV antibodies and inhibition of AAV transduction in primates (Li et al. 2012). Given this, we estimated the levels of AAV2/9 antibodies in the macaques before and after vaccination. First, we randomly selected seven macaques, including two macaques each in the low- and middle-dose groups (group 2) and three macaques in the high-dose group (group 1) to evaluate their pre-existing levels of AAV2/9 antibodies. All tested macaques were AAV2/9 ­antibody-positive (Fig. 2B and 2C), suggesting that AAV2/9 antibodies may commonly exist in this species. Interestingly, the AAV2/9 antibodies levels did not change significantly from 56 to 217 dpv compared with day 0, even in the high-dose AAV-SRBD macaques. These results suggest the pre-existence of AAV2/9 antibodies in macaques before immunization, and that intramuscular injection of the AAV-based vaccine did not boost AAV antibody levels.

**Figure 2.**
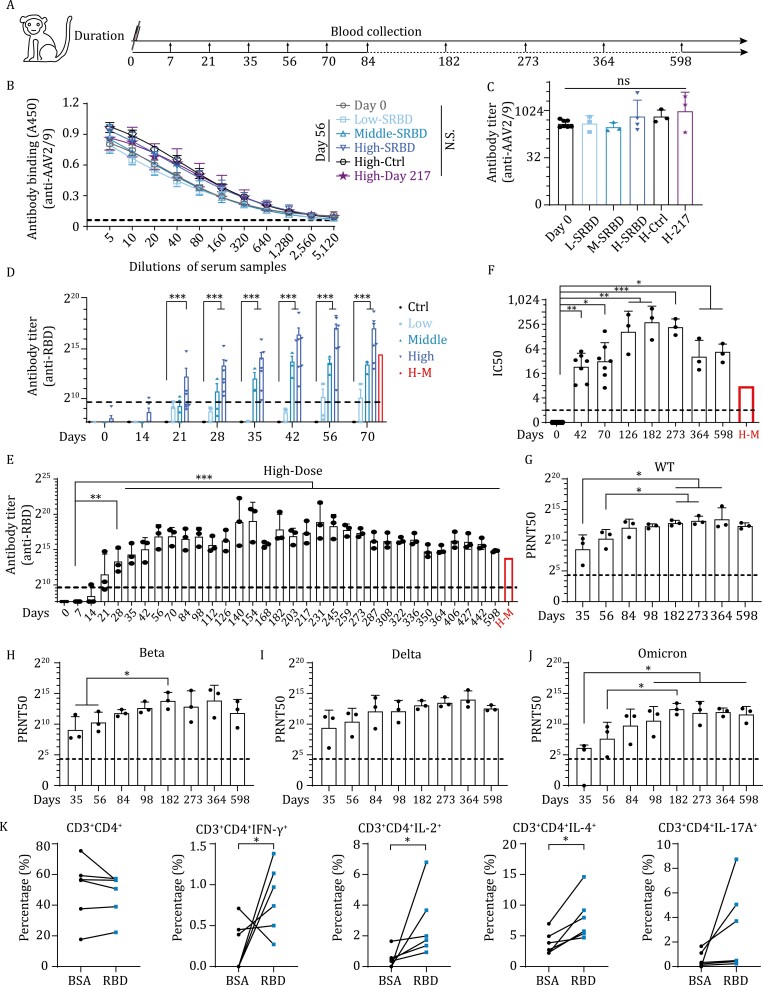
Single-dose SRBD vaccine elicits long-lasting and high-level humoral responses in rhesus macaques. (A) Experimental strategy of AAV-SRBD vaccine analysis in rhesus macaques. (B) ELISA of AAV2/9 antibodies in macaque serum. (Day 0: serum collected before intramuscular injection; Low-/Middle-/High-SRBD: 56 dpv serum of low-/middle-/high-dose SRBD vaccine; High-Ctrl: 56 dpv serum of high-dose AAV-CAG-GFP control; High-Day 217: 217 dpv serum of high-dose SRBD vaccine; *n* = 3 macaques in Low-SRBD, Middle-SRBD, High-Ctrl, High-Day 217 groups; *n* = 7 macaques in Day 0 group; *n* = 4 macaques in High-SRBD group). (C) Quantitative analysis of AAV2/9 antibody titer of ELISA data in B. (D) Quantitative analysis of RBD antibodies titer calculated by ELISA from 0 to 70 dpv in low/middle/high-dose groups and AAV-CAG-GFP control macaques (*n* = 3 macaques in Ctrl, low-, and middle-dose group, respectively; *n* = 7 macaques in high-dose group). Mean RBD antibody titer of H-M is represented by red bar. The cutoff for the positive antibody titer is presented by black dot line. (E) Quantitative analysis of RBD antibody titer calculated by ELISA of RBD antibodies from 0 to 598 dpv in high-dose macaques of group 1 (*n* = 3 macaques in each group). Mean RBD antibody titer of H-M is represented by red bar. The cutoff for the positive antibody titer presented by black dot line. (F) Quantitative analysis of RBD NAb IC50 levels calculated by competitive ELISA from 0 to 598 dpv in high-dose macaques (*n* = 7 macaques in day 0, 42, and 70 group; *n* = 3 macaques in other groups). Mean IC50 of H-M is represented by red bar. The cutoff for the positive IC50 presented by black dot line. (G) Serum PRNT50 level in group 1 high-dose macaques from 35 to 598 dpv calculated using plaque reduction neutralization test in Vero E6 cells against wild-type SARS-CoV-2 (*n* = 3 macaques in each group). The cutoff for the positive antibody PRNT50 presented by black dot line. (H) Serum PRNT50 level in group 1 high-dose macaques from 35 to 598 dpv calculated by plaque reduction neutralization test in Vero E6 cells against Beta variant (*n* = 3 macaques in each group). The cutoff for the positive antibody PRNT50 presented by black dot line. (I) Serum PRNT50 level in group 1 high-dose macaques from 35 to 598 dpv calculated by plaque reduction neutralization test in Vero E6 cells against Delta variant (*n* = 3 macaques in each group). The cutoff for the positive antibody PRNT50 presented by black dot line. (J) Serum PRNT50 level in group 1 high-dose macaques from 35 to 598 dpv calculated by plaque reduction neutralization test in Vero E6 cells against Omicron variant (*n* = 3 macaques in each group). The cutoff for the positive antibody PRNT50 presented by black dot line. Data (G to J) were obtained from one experiment. Each serum was tested three times as technical replicates. (K) Percentage of CD3^ + ^CD4^+^, CD3^ + ^CD4^ + ^IFN-γ^+^, CD3^ + ^CD4^ + ^IL-2^+^, CD3^ + ^CD4^ + ^IL-4^+^, and CD3^ + ^CD4^ + ^IL-17A^ + ^cells in blood of high-dose rhesus macaques activated by BSA or RBD peptide (*n* = 6 macaques in each group). Values are means ± s.e.m. or geometric mean + geometric standard deviation for antibody titer and PRNT50. *P < 0.05; **P < 0.01; ***P < 0.001.

Even though AAV2/9 antibodies pre-existed in the macaques, the seroconversion rate (antibody titer > 800) reached 100% on 35 dpv in the high- (7/7) and middle-dose (3/3) macaques, but only 33.3% (1/3) at 56 dpv in the low-dose macaques (Fig. 2D). Accordingly, the AAV-SRBD vaccine demonstrated good immunogenicity in the high- and middle-dose macaques, but not in the low-dose macaques, as tested by enzyme-linked immunosorbent assay (ELISA) (Figs. 2D and S2C) and competitive ELISA (Fig. S2D–G). The SRBD NAbs in the high- and middle-dose macaques effectively inhibited interactions between the RBD and ACE2, and the efficacy was better than that of mixed sera from convalescent COVID-19 patients with severe disease (H-M) (Fig. S2D–G) (Ma et al. 2021). These results suggest that AAV-SRBD induced robust humoral responses in NHPs, and vaccine efficacy appeared to be highly dose-dependent (*P *= 0.0293 in Fig. 2D; *P* = 0.0221 in Fig. S2E; *P* = 0.0090 in Fig. S2G by one-way ANOVA). To assess the long-term humoral immune response of the AAV vaccine, we also monitored the SRBD antibody levels in the high-dose macaques (group 1) from days 0 to 598 dpv (Fig. 2E). Results indicated that SRBD antibodies emerged on 21 dpv in the high-dose macaques and remained at a high level until 598 dpv, with an average titer higher than found in the H-M. The absorbance at 450 nm (A450) values demonstrated that the binding of RBD to SRBD antibodies increased with time but decreased slightly at 364 dpv and 598 dpv (Figs. 2E and S2H), as found for the inhibitory ability of NAbs (Figs. 2F and S2I). However, the inhibition rate was still higher than that in the H-M samples at 598 dpv. These results indicate that AAV-SRBD triggers a robust and long-lasting humoral response after a single-dose of vaccine, and that pre-existing AAV2/9 antibodies do not interfere with the vaccine immunity.

The SRBD NAbs from high-dose macaques (group 1) were further measured using the plaque reduction neutralization test (PRNT), which is the gold-standard for determining immune protection. As the SARS-CoV-2 variants especially Delta and Omicron variants widely spread worldwide, we evaluated the SRBD NAbs against authentic wild-type SARS-CoV-2, Beta, Delta, and Omicron variants using standard PRNT assay (Figs. 2G–2J and S3). The 50% reduction in plaque count (PRNT50) value of the SRBD NAbs increased from 35 to 182 dpv, and remained at a high level (geometric mean of PRNT50 > 1:2,048) in all macaques after 98 dpv against the wild-type SARS-CoV-2 virus. The PRNT50 values against the Beta and Delta variants were similar to the value against the wild-type SARS-CoV-2 virus (geometric mean of PRNT50 > 1:2,048) after 98 dpv. However, geometric mean of PRNT50 against the Omicron variant was lower than that against wild-type SARS-CoV-2 virus, but all sera tested still kept in high level, showing a PRNT50 from 893 to 11,112 after 182 dpv. These data together illustrated that the AAV-SRBD vaccine induced high and effective NAbs against the wild-type SARS-CoV-2, Beta, Delta, and Omicron variants in rhesus macaques, and the AAV-SRBD vaccine provided efficient cross neutralization against major SARS-CoV-2 variants. Based on these results, it is reasonable to believe that our vaccine is broad-spectrum and could provide protection for future emerging variants.

To assess the antigen-specific T cell responses to the AAV-SRBD vaccine, we used the RBD peptide pool to stimulate peripheral blood mononuclear cells (PBMCs) collected from high-dose macaques at 35 dpv. Compared to the bovine serum albumin (BSA) control, the percentages of the CD4^ + ^IFN-γ^+^, CD4^ + ^IL-2^+^, CD4^ + ^IL-4^+^, and CD8^ + ^IL-4^ + ^T cells increased under RBD peptide pool stimulation (Fig. 2K and Fig. S4). These results indicate that RBD-specific Th1 (IFN-γ^+^ and IL-2^+^) cell and Th2 (IL-4^+^) cell responses in PBMCs can be activated by stimulation of the RBD peptide pool after vaccination.

The toxicity of the SRBD vaccine was further evaluated in rhesus macaques. As of 598 dpv, no deaths, impending deaths, or significant abnormalities in clinical physiology were found in any macaque. Widely analyzed pathological indicators also showed that lymphocyte subgroup (CD20^+^, CD3^+^, CD3^ + ^CD4^+^, and CD3^ + ^CD8^+^) distribution was normal before and after intramuscular injection (Fig. S5, stimulation with cocktail) by 70 dpv. These results strongly suggest that the AAV-SRBD vaccine is safe and does not trigger severe inflammation. Other pathological indicators in blood, i.e., white blood cell (WBC), monocyte (MONO), neutrophil (NEUT), eosinophil (EO), basophil (BASO), lymphocyte (LYMPH), red blood cell (RBC), and platelet (PLT) counts, were also normal before and after immunization with different doses of the AAV vaccine (Fig. S6). These results suggest that the vaccine did not cause blood toxicity after injection. On the other hand, we found that the AAV was expressed in the liver of C57BL/6J mice (Fig. S1C), which was also supported by other studies (Greig et al. 2014). Similarly, we detected SRBD expression in the livers of the high-dose macaques, but very little in the low-dose group. To exclude the hepatotoxicity of the AAV-SRBD vaccine, we examined the hepatic function of the immunized macaques. Results showed no significant change of the levels of Alkaline phosphatase (ALP), Total bilirubin (TBIL), Alanine aminotransferase (ALT), Aspartate aminotransferase (AST) before and after vaccination. Thus, although AAV gene expression was detected in the liver of the high-dose macaques, it did not appear to trigger liver dysfunction and inflammation (Fig. S7).

Currently, several SARS-CoV-2 variants are of worldwide concern. Thus, we investigated whether the SRBD vaccine shows efficacy against variants such as B.1.1.7 (Alpha), B.1.351 (Beta), P.1/P.2 (Gamma), B.1.617.2 (Delta), B.1.617.1/3 (Kappa), and C.37 (Lambda). To test the inhibitory ability of SRBD NAbs against different SARS-CoV-2 variants, we generated several RBDs in different variants, i.e., B.1.1.7, B.1.351 and P.1/P.2, B.1.617.1/2/3, and C.37. Based on competitive ELISA, the macaque serum effectively inhibited interactions between the RBD mutants and ACE2 (Fig. S8). Therefore, the SRBD NAbs appear to offer long-term inhibitory activity against SARS-CoV-2 variants. These results indicate that the SRBD vaccine has the potential to block infection from SARS-CoV-2 variants.

In conclusion, we developed a single-dose vaccine that can provide long-term protection against SARS-CoV-2. Our results showed that SRBD is more thermostable than RBD. The AAV-SRBD vaccine could induce good seroconversion rate in both NIH and C57BL/6J mice. This vaccine overcomes the multiple injection requirement of current vaccines and provides high-level and long-lasting RBD NAbs. Moreover, the presence of pre-existing immunity to AAV2/9 here did not restrict the delivery or efficacy of AAV-SRBD. We suspect that AAV rapidly enters the cells and AAV antibody titer is relatively low in muscles, allowing AAV-SRBD to overcome the inhibition of AAV antibodies. A potent immune response to AAV-ovalbumin was observed when AAV was administered intravenously but not when administered intramuscularly (Daya and Berns 2008). Importantly, the SRBD vaccine provides cross neutralization against emerging variants. Further studies on wild-type SARS-CoV-2 and Omicron variant challenge in macaques are in progress to explore the protective immunity of the AAV-SRBD vaccine. Another study reported on two AAV-based vaccines that demonstrate long NAb durability in mice and NHPs with a single injection, further supporting the safety and efficacy of AAV-based vaccines (Demminger et al. 2020). Thus, this vaccine shows great potential for the control of COVID-19.

## Supplementary Material

pwac020_suppl_Supplementary_Material

## Footnotes

We thank all colleagues from the National Biosafety Laboratory (Wuhan), Chinese Academy of Sciences, China, for their support during the study. We thank the Center for Instrumental Analysis and Metrology and Biosafety Level 3 Laboratory, Wuhan Institute of Virology, Wuhan, China. We thank Professor Yifeng Zhou, Professor Li Bai, Dr. Fei Liu and their lab members for discussion and assisting in the design of the experiments. We thank Jia Wu, Jun Liu and Hao Tang from Wuhan Institute of Virology for the management of BSL-3 facility, where all the authentic ­SARS-CoV-2 experiments were conducted.

This work was supported by Strategic Priority Research Program of the Chinese Academy of Sciences (XDB39000000 to T.X.), National Natural Science Foundation of China (81925009 to T.X., 81790644 to T.X., 81900855 to M.Z., 82000941 to D.T.), Jack Ma Foundation (2019-nCoV), CAS Project for Young Scientists in Basic Research (YSBR-013) and Fundamental Research Funds for the Central Universities (WK5290000001 to Y.C., WK5290000002 to Y.Y., WK2090050048 to M.Z., WK2070000174 to M.Z.). The study was also supported by the Anhui Provincial Natural Science Foundation (1808085MH289 to M.Z.). Joint Laboratory of Innovation in Life Sciences from the University of Science and Technology of China (USTC) and Changchun Zhuoyi Biological Co. Ltd.

T.X., T.J., Y.C., M.Z., and D.T. are inventors of a pending patent related to this work filed by the University of Science and Technology of China (no. 202010903143.9, filed on 3 December 2020). The authors declare no other competing interests.

All institutional and national guidelines for the care and use of laboratory animals were followed. All procedures were conducted in accordance with the Principles for the Ethical Treatment of Animals approved by the Animal Care and Use Committee at the University of Science and Technology of China (Animal ethics number: 202006220919000464981).

All data needed to evaluate the conclusions of the paper can be found in the manuscript and/or Supplementary Material. Additional data related to this paper may be requested from the lead contact Professor Tian Xue.

T.X., Z.Y., T.J., and Z.Q. conceived the project and designed the experiments. D.T. and M.Z. developed the vaccines, tested their immunogenicity and safety, and wrote the manuscript. Y.Y. constructed the SARS-CoV-2 variants, coated the ELISA plates, and performed flow cytometry. H.X. and H.Z. performed the neutralizing antibody assay in Vero E6 cells. H.T., W.Z., M.L., Y.W., H.M., X.H., W.L., Y.C., Y.Y., Y.Y., K.L., S.S., Y.L., G.G., W.G., Y.P., S.C., J.R., J.Z., J.M., Q.Z., Y.Z., L.L., C.S., and K.Z. worked on data collection, analysis, and discussion. All authors edited and proofread the manuscript.
