# Supplementary material for: Single-dose AAV-based vaccine induces a high level of neutralizing antibodies against SARS-CoV-2 in rhesus macaques
Source: Protein Cell. 2022 Jul 15;14(1):69–73. doi: 10.1093/procel/pwac020 (PMC9871966; doi:10.1093/procel/pwac020)
Supplement: pwac020_suppl_Supplementary_Material [file pwac020_suppl_supplementary_material.docx]

**Materials and Methods**

**Animal husbandry samples**

C57BL/6J mice, NIH mice, and rhesus macaques (*Macaca mulatta*) were maintained at 25 °C on a 12 h: 12 h light:dark cycle in an animal room at the University of Science and Technology of China. All procedures were conducted in accordance with the Principles for the Ethical Treatment of Animals approved by the Animal Care and Use Committee at the University of Science and Technology of China (Animal ethics number: 202006220919000464981). The macaques received a single-dose immunization (1 mL) of SRBD (1 × 10^12^ vg/macaque (high-dose, 4 males and 3 females), 1 × 10^11^ vg/macaque (middle-dose, 2 males and 1 female), or 1 × 10^10^ vg/macaque (low-dose, 1 male and 2 females) or AAV-CAG-GFP (1 × 10^12^ vg/macaque; control, 1 male and 2 females) by intramuscular injection. Mice received a single-dose immunization (20 µL) of SRBD, RBD, or AAV-CAG-GFP (1 × 10^11^ vg/mouse, males and females). For immunohistochemical analysis, mice received different doses (1 × 10^11^, 1 × 10^10^, and 1 × 10^9^ vg/mouse, males and females) of AAV-CAG-GFP.

**AAV packaging**

The AAV vaccines were packaged in HEK-293T cells. In brief, 10 μg of pHelper vector, 5 μg of AAV2/9 vector, and 5 μg of pITR vector (RBD/SRBD/GFP, Figure S1A) were transfected into a 10-cm diameter dish with HEK-293T cells by polyethylenimine (PEI) (PolyScience, Niles, USA). The HEK-293T cells were cultured with Dulbecco’s Modified Eagle Medium (DMEM, Gibco, 11965-092) with 10% fetal bovine serum (FBS) (Gibco, 16000-044) at 37 °C. The supernatant of the HEK-293T cells was harvested at days 3 and 6 after transfection. The supernatant was concentrated with Ultra-15 Centrifugal Filters (Millipore, UFC905024) and then gradient-purified with 15% to 60% Optiprep (Sigma, D1556). The AAV vaccine was harvested and washed using phosphate-buffered saline (PBS) six times, then diluted in 150 μL of PBS. The titers of the AAV vaccines were calculated by quantitative real-time polymerase chain reaction (qRT-PCR). The AAV vaccines were stored at −80 °C.

**Electron microscope (EM) sample preparation**

The AAV vaccines (1 × 10^12^ vg/mL) were added to carbon-coated copper grids previously glow-discharged at low air pressure and stained with 2% uranyl acetate for 90 seconds. The EM was operated at an acceleration voltage of 120 kV. Images were recorded using a Tecnai G2 Spirit 120kV EM at 23 000× magnification.

**Protein expression and purification**

The methods for purifying the SARS-CoV-2 RBD [amino acid (AA) 321–591], SARS-CoV-2 RBD variants, and human ACE2 extracellular domain (AA 19 to 615) followed previous research (Ma et al., 2021). In brief, target genes were inserted into the pTT5 vector, which contains a IFNA1 signal peptide at the N-terminus and a TEV enzyme site connected to the human IgG1 Fc at the C-terminus. The expression vectors were then transiently transfected into HEK-293F cells using polyethyleneimine (Polyscience). After 3 days, the supernatant was collected by centrifugation at 5 000 ×*g* for 15 min at 4 °C. About ¼ volume of 1 × PBS was added to adjust the pH of the supernatant. The supernatant was then loaded onto the protein A column and the target protein was eluted with 0.1 M acetic acid on ÄKTA pure (GE Healthcare). The collected protein was added to 1 mM edetate disodium (EDTA), 5 mM dithiothreitol (DTT), and tobacco etch virus (TEV) protease to remove Fc on a shaker in a 4 °C freezer. After dialysis in 1 × PBS, tandem protein A and nickel columns were used for further purification. Both Fc and undigested protein were loaded onto the protein A column and TEV (6 × His tag) was loaded onto the nickel column. The target protein was collected during flow through.

**Thermal stability analysis**

To compare the thermal stability of RBD and SRBD, circular dichroism (CD) spectra were acquired on a Chirascan Spectrometer (Applied Photophysics, Leatherhead, UK). Prior to CD measurements, the sample buffers were changed to PBS and the protein concentration was adjusted to 0.5 mg/mL, as determined by its absorbance at 280 nm. For thermal titration, CD spectra were acquired from 20 to 95 °C with temperature steps of 5 °C and wavelengths between 180–260 nm. The CD signals at 222 nm were used to characterize structural changes during thermal titration. The data were fitted by the GraphPad Prism 8.0 software to calculate the T_m_ values.

**Vaccine immunogenicity analysis**

The C57BL/6J and NIH mice (8 weeks old; male and female; 20–25 g body weight for C57 mice and 30–35 g body weight for NIH mice) were randomly divided into five groups (five mice per group). The mice were intramuscularly injected with RBD or SRBD AAV vaccines or the AAV-CAG-GFP control at a dose of 1 × 10^11^ vg (20 μL). The macaques were randomly divided into four groups (seven macaques in high-dose group and three macaques in other groups) and intramuscularly injected with 1 mL of SRBD vaccine (1 × 10^12^, 1 × 10^11^, and 1 × 10^10^ vg/macaque for high/middle/low dose, respectively) or AAV-CAG-GFP control (1 × 10^12^ vg/macaque). Blood was collected from macaques before immunization and at every 7 days before day 42 after injection and every 14 or 21 days after day 42.

**ELISA and** **competitive ELISA**

Nunc MaxiSorp plates were coated with 3 μg/mL recombinant RBD, P.1/P.2 RBD, B.1.1.7 RBD, B.1.617RBD, B.1.351 RBD, or 3 μg/mL AAV9 at 4 °C overnight. After washing four times with PBS (3 min each time), the plates were blocked with 5% non-fat milk in PBS at room temperature for 2 h. Serially diluted serum (5% non-fat milk in PBST (PBS with 0.1% Tween-20) for ELISA or 5% non-fat milk in PBST with 15 nM biotin-ACE2-TEV-Fc for competitive ELISA) was added to the plates, which were then incubated at room temperature for 1 h. After washing three times with PBST (3 min each time), horseradish peroxidase (HRP)-conjugated goat anti-mouse IgG (Sangon Biotech, D110087, mouse serum, ELISA), rabbit anti-monkey IgG (Cellwaylab, C020217, macaque serum, ELISA), or HRP-conjugated streptavidin (Beyotime, A0303, competitive ELISA) were added, followed by incubation at room temperature for 1 h. For macaque serum ELISA, HRP-conjugated goat anti-rabbit IgG (Sangon Biotech, D110058) was added, followed by incubation at room temperature for 1 h. After washing three times with PBST (3 min each time), TMB substrate (Beyotime, P0209) was added for 8 min, then stopped by 1 M H_2_SO_4_. Absorbance at 450 nm was measured with a microplate reader.

Antibody titer was calculated as the dilution of the serum that induces an A450 value twice that of the A450 value of the negative control. Dotted lines in Figure 2B and Figure S2B and H represent the A450 value of the negative control. The Dotted line in the Figure 2D-F and Figure S2F represents the cutoff of RBD antibody titer and IC50 of the macaques’ sera (Cutoff defined as 4-fold higher in antibody titer or IC50 compared to the negative baseline; because of the antibody titer of negative baseline could not been calculated by the Elisa curve as it is much lower than the first dilution of sera, so we use the first dilution (1:200 in Elisa and 1:1 in competitive ELISA) as the antibody titer of negative baseline ).

**Virus and cells**

Vero E6 cells were maintained in Dulbecco's modified Eagle's medium (DMEM, Gibco) supplemented with 10% fetal bovine serum (FBS, ExCell Bio) and 1% penicillin-streptomycin (Gibco) at 37°C under a 5% CO2 atmosphere. The SARS-CoV-2 WIV04 strain was originally isolated from a COVID-19 patient in 2019 (GISAID, accession no. EPI_ISL_402124)(Zhou et al., 2020); Beta variant (NPRC2.062100001) was kindly provided by Chinese Center for Disease Control and Prevention(Li et al., 2021), and Delta variant (B.1.617.2; GWHBEBW01000000) by Prof. Hongping Wei; Omicron variant (B.1.1.529; BA.1) was isolated from a throat swab of a patient from Hong Kong by the Institute of Laboratory Animal Sciences, Chinese Academy of Medical Sciences (CCPM-B-V-049-2112-18). All processes in this study involving authentic SARS-CoV-2 were performed in a BSL-3 facility.

**Plaque reduction neutralization test in Vero E6 cells**

Briefly, antibodies were serially diluted with DMEM containing 2.5% FBS, and mixed with equal volume of virus suspension and incubated at 37°C for 1 h. The mixture was added to Vero E6 monolayer cells in 24-well plates and incubated for another 1 h, and the inoculate was replaced with DMEM containing 2.5% FBS and 0.9% carboxymethyl-cellulose. The plates were fixed with 8% paraformaldehyde and stained with 0.5% crystal violet 3 days later. Plaque reduction neutralizing titer was calculated using the “inhibitor vs normalized response (Variable slope)” model in the GraphPad Prism 8.0 software. The Cut-off value is calculated by the negative control (geometric mean + 3 times of geometric standard deviation).

**Reverse Transcription-Polymerase Chain Reaction (RT-PCR)**

Total RNA was extracted from organs with Trizol reagent (Invitrogen, 15596026) and a PrimeScript RT Reagent Kit (Takara, RR037A). Forward and reverse primers were designed to target RBD sequence (forward: 5’-GTGTACGCCTGGAATCGGAA-3’ and reverse: 5’-GATCTCGGTGCTGATGTCCC-3’),

mouse GAPDH sequence (forward: 5’- AGGTCGGTGTGAACGGATTTG -3’ and reverse: 5’- TGTAGACCATGTAGTTGAGGTCA -3’),

macaque GAPDH sequence (forward: 5’- AGAAGACTGTGGATGGCCCCT-3’ and reverse: 5’- TACACGACAAGGTGGGGCTCC-3’),

CAG sequence (forward: 5’- AACGCCAATAGGGACTTTCCATTGA-3’ and reverse: 5’- ATGGGGAGAGTGAAGCAGAACG -3’).

**Hematoxylin-eosin (H&E) staining**

Mice were anesthetized with sodium pentobarbital (40 mg/kg, intraperitoneal injection), then perfused with PBS and fixed in 4% paraformaldehyde (PFA). Organs were post-fixed in 4% PFA overnight at 4 °C, then dehydrated in 15% and 30% sucrose, respectively. Organs were sectioned at a thickness of 10 μm for H&E staining with a freezing microtome. Isopropanol (500 μL) was added to the slices and incubated at room temperature for 1 min. The slices were air dried, stained with hematoxylin (Agilent, S330930-2, 1 mL), and incubated at room temperature for 7 min. The slices were then washed 10 times (10 s each time) with ultrapure water, followed by the addition of bluing buffer (Agilent, CS70230-2, 1 mL) and incubation at room temperature for 2 min. After washing five times with ultrapure water (10 s each time), eosin mix (Sigma, HT110216, 1 mL) was added, and the sections were incubated at room temperature for 1 min.

**Immunohistochemical analysis**

Organs (muscle, liver, heart, lung, spleen, kidney and brain) were sectioned (40-μm thick) for immunohistochemical analysis with a freezing microtome. After washing with PBS three times (5 min each time), the slices were blocked with 3% bovine serum albumin (BSA) and 0.1% Triton-X100 in PBS for 1 h at room temperature. The slices were then stained using 1:1 000 anti-GFP antibody (Earthox, E002030-02) in blocking buffer overnight at 4 °C. Slices were washed with PBS three times (15 min each time) and incubated with secondary antibodies Alexa Fluor 488 donkey anti-mouse IgG (1: 1000, Thermo Scientific, A21202) for 2 h at room temperature. DAPI (1:1000, Thermo Scientific, D3571) was used to stain cell nuclei. Confocal images were captured using a Leica microscope.

**Peripheral blood mononuclear cells (PBMCs), exocellular and intracellular staining, and flow cytometry**

Blood samples from macaques were collected in EDTA-2K tubes. Ficoll medium (3 mL, Solarbio, P4350) was first added into a 15-mL tube, followed by blood (3 mL) and density gradient centrifugation at 400 ×*g* for 20 min at room temperature (ACC/DEC: 6/2). The plasma was then collected and stored at −80 °C. CELLSAVING buffer (Xinsaimei, C40100) was used to resuspend the PBMCs after thorough washing with PBS, with the cells then stored at −80 °C.

The frozen cells were resuspended and washed in RPMI medium 1640 (Gibco). Anti-CD3 (BD Biosciences, 557705), anti-CD4 (BioLegend, 357423), anti-CD8 (BioLegend, 301007), and anti-CD20 (BioLegend, 302310) antibodies were added to the cells for staining for 30 min in the dark on ice. After washing with PBS (30 s), the cells were tested on a BD FACSVerse flow cytometer. The PBMCs were also resuspended in RPMI medium 1640, with a cocktail (BD Biosciences, 550583) added to activate the cells at 37 °C for 4 h. Cells were washed with 1 × PBS (30 s) and stained on ice in the dark for 30 min with anti-CD3 (BD Biosciences, 557705), anti-CD4 (BioLegend, 357423), and anti-CD8 (BioLegend, 344714). The cells were then fixed and permeabilized using a Cytofix/Cytoperm Soln Kit (BD Biosciences, 554714). Afterwards, cells were stained with anti-IFN-γ (BioLegend, 502526), anti-TNF-α (BioLegend, 502930), and anti-IL-10 (BioLegend, 501420) antibodies and incubated on ice in the dark for 1 h. After washing with PBS (30 s), the cells were tested on a BD FACSVerse flow cytometer. FlowJo v10 software was used for data analysis.

**T cell stimulation**

The frozen cells were resuspended and washed with RPMI medium 1640 (Gibco), and then 1 × 10^6^ cells per well were transferred to a sterile 96-well plate and cultured with complete medium (RPMI 1640 supplemented with 10% FBS, penicillin, streptomycin). Then, 2 μg/ml RBD peptide pool (Sino Biological, PP002-A) was added to the medium for 18-h stimulation. Cells were stimulated with BSA as the negative control. Co-stimulators, i.e., 2 μg/ml anti-human CD28 (BD Biosciences, 1050151) and 100 KU/ml (sigma, I7908-10KU), were then added to the medium. In the last 6 h of stimulation, 5 μg/ml brefeldin A (BioLegend, 420601) and 2.5 μg/ml monensin (Selleckchem, S2324) were added to the medium. The cells were stimulated with leukocyte activation cocktail (BD Biosciences, 550583) as a positive control.

**Flow cytometry**

The stimulated cells were washed with PBS containing 2% FBS. The cells were then stained on ice in the dark for 45 min with anti-CD3 (BD Biosciences, 557705), anti-CD4 (BioLegend, 317417), and anti-CD8 (BioLegend, 344712). The cells were fixed and permeabilized using a Cytofix/Cytoperm Kit (BD Biosciences, 554714). Afterwards, cells were stained with anti-IFN-γ (BioLegend, 502526), IL-2 (BioLegend, 500306), IL-4 (BioLegend, 500826), and IL-17A (BioLegend, 512329) antibodies and incubated on ice in the dark for 1 h. Cells were washed and resuspended with PBS and detected on the BD FACSVerse flow cytometer. FlowJo v10 was used for data analysis.

**Statistical analyses**

All data are presented as means ± standard error of the mean (SEM), except for the titers of NAbs, which were quantified using geometric mean + geometric standard deviation. Student’s *t*-test and paired *t-*test were used to determine the statistical significance of differences between two groups. One-way analysis of variance (ANOVA) and two-way ANOVA were used to determine statistical significance for different dose groups and the curve graphs, respectively. Quantification graphs were analyzed using GraphPad Prism v8 (GraphPad Software). *: *P* < 0.05; **: *P* < 0.01; ***: *P* < 0.001.

**References**

Li, T., Han, X., Gu, C., Guo, H., Zhang, H., Wang, Y., Hu, C., Wang, K., Liu, F., Luo, F., et al. (2021). Potent SARS-CoV-2 neutralizing antibodies with protective efficacy against newly emerged mutational variants. Nat Commun *12*, 6304. 10.1038/s41467-021-26539-7.

Ma, H., Zeng, W., Meng, X., Huang, X., Yang, Y., Zhao, D., Zhou, P., Wang, X., Zhao, C., Sun, Y., et al. (2021). Potent Neutralization of SARS-CoV-2 by Hetero-bivalent Alpaca Nanobodies Targeting the Spike Receptor-Binding Domain. J Virol. 10.1128/JVI.02438-20.

Zhou, P., Yang, X.L., Wang, X.G., Hu, B., Zhang, L., Zhang, W., Si, H.R., Zhu, Y., Li, B., Huang, C.L., et al. (2020). A pneumonia outbreak associated with a new coronavirus of probable bat origin. Nature *579*, 270-273. 10.1038/s41586-020-2012-7.

**Table S1. Summary of rhesus macaques for vaccination**

| Group | Animal NO. | Vaccination Dose | Gender | Age (Years) | Sacrificed time （dpv） | Experiment |  |
| --- | --- | --- | --- | --- | --- | --- | --- |
|  |  |  |  |  |  |  |  |
| 1 | 1H-1 | 1×10^12^vg AAV-SRBD | M | 6 | Live | Elisa, competitive-ELISA, authentic virus neutralization, flow cytometry |  |
|  | 1H-2 |  | M | 7 |  |  |  |
|  | 1H-3 |  | F | 5 |  |  |  |
| 2 | 2H-1 | 1×10^12^vg AAV-SRBD | F | 5 | 70 | Elisa, competitive-ELISA, body weight, pathological indicators in blood, hepatic function, flow cytometry |  |
|  | 2H-2 |  | M | 6 |  |  |  |
|  | 2H-3 |  | M | 6 |  |  |  |
|  | 2H-4 |  | F | 5 |  |  |  |
|  | 2M-1 | 1×10^11^vg AAV-SRBD | M | 7 | 70 |  |  |
|  | 2M-2 |  | M | 5 |  |  |  |
|  | 2M-3 |  | F | 6 |  |  |  |
|  | 2L-1 | 1×10^10^vg AAV-SRBD | F | 6 | 70 |  |  |
|  | 2L-2 |  | M | 7 |  |  |  |
|  | 2L-3 |  | F | 5 |  |  |  |
|  | 2C-1 | 1×10^12^vg AAV-GFP | F | 5 | 70 |  |  |
|  | 2C-2 |  | F | 6 |  |  |  |
|  | 2C-3 |  | M | 6 |  |  |  |

**
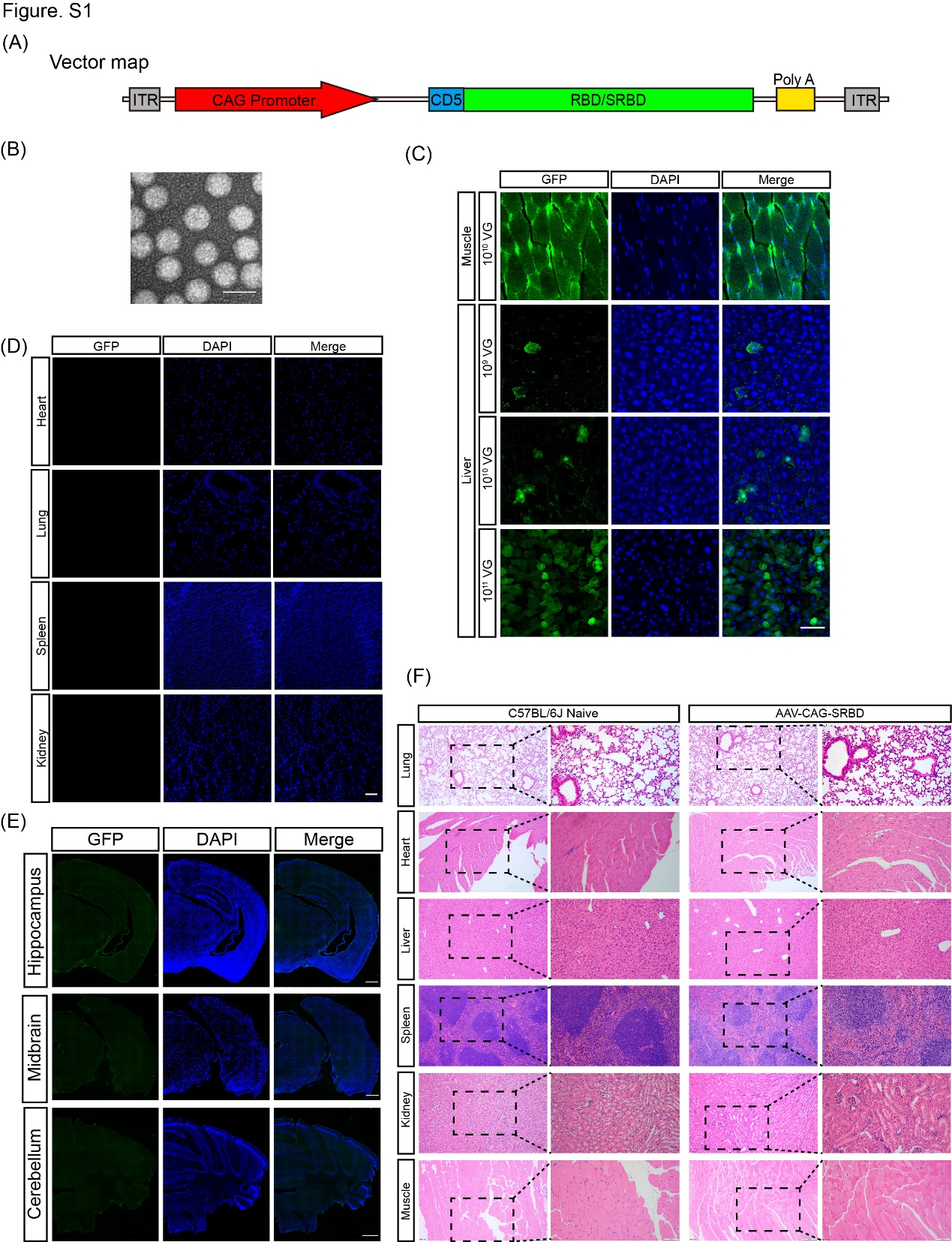
Figure S1. Expression of AAV2/9- delivered GFP in the major organs of mice**

(A) Schematic representation of recombinant genome of AAV-SRBD and AAV-RBD vaccine candidates. CAG, CMV immediate enhancer/β-actin (CAG) promoter; ITR, inverted terminal repeat; CD5, CD5 signaling peptide.

(B) Images of AAV-SRBD particles by electron microscopy analysis (scale bar: 50 nm).

(C) Expression of GFP in muscle and liver from low/middle/high-dose AAV-GFP C57BL/6J mice at 42 dpv (scale bar: 50 μm).

(D) Expression of GFP in heart, lung, spleen, and kidney of high-dose AAV-GFP-injected C57BL/6J mice at 42 dpv (scale bar: 50 μm).

(E) Expression of GFP in brain of high-dose AAV-GFP-injected C57BL/6J mice at 28 dpv (scale bar: 500 μm).

(F) H&E-stained lung, heart, liver, spleen, kidney, and muscles of naïve (Control group) and AAV-SRBD-injected C57BL/6J mice at 42 dpv. Tissues were all normal in morphology (scale bar: 200 µm in the left row of each group and 100 µm in the right row of each group).

**
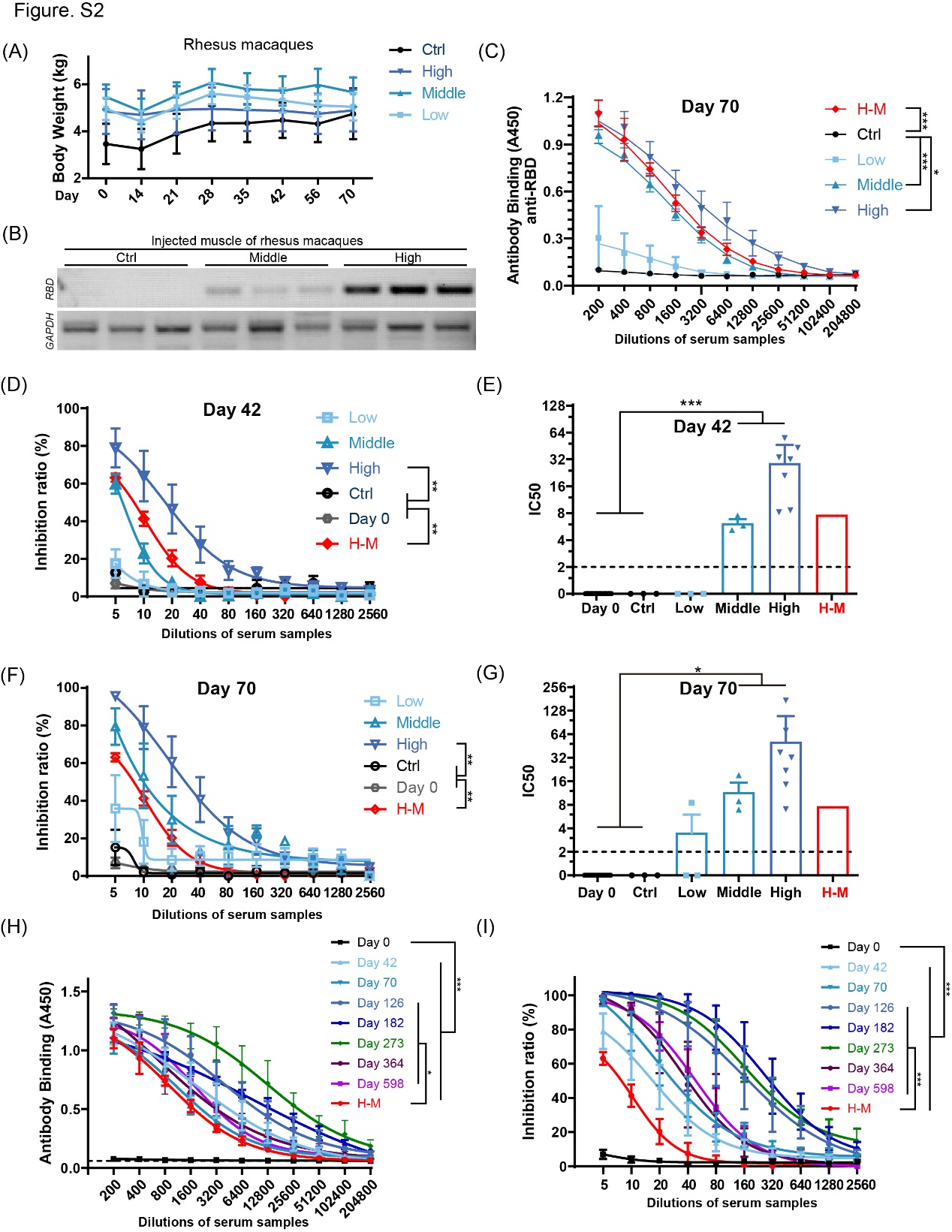
Figure S2. Quantitative analysis of humoral responses in AAV-SRBD vaccine-injected rhesus macaques.**

(A) Weekly monitoring of body weight from 0 to 70 dpv in different dose macaques in group 2 (Ctrl: macaques intramuscularly injected with high-dose of AAV-CAG-GFP control; Low/Middle/High: macaques intramuscularly injected with low/middle/high-dose of AAV-SRBD vaccine; n = 3 macaques in Ctrl, Low, and Middle groups; n = 4 macaques in High group).

(B) RT-PCR data of RBD expression in middle- and high-dose AAV-SRBD- or AAV-CAG-GFP-injected macaques (n = 3 mice in each group).

(C) ELISA of RBD antibodies from macaque serum at 70 dpv (n = 3 macaques in Ctrl, Low, and Middle groups; n = 3 repeats in H-M group; n = 7 macaques in High group).

(D) Competitive ELISA of inhibition of SARS-CoV-2 RBD-hACE2 interaction by macaque serum at 42 dpv (n = 3 macaques in Ctrl, Low, and Middle groups; n = 3 repeats in H-M group; n = 7 macaques in High group; n = 16 macaques in Day 0 group).

(E) Quantitative analysis of RBD NAb half maximal inhibitory concentrations (IC50) between ACE2 and RBD, calculated by competitive ELISA at 42 dpv (n = 3 macaques in Ctrl, low, middle groups; n = 7 macaques in high-dose group; n = 16 macaques in Day 0 group). Mean IC50 of H-M is represented by red bar. The cutoff for the positive IC50 presented by black dot line.

(F) Competitive ELISA of inhibition of SARS-CoV-2 RBD-hACE2 interaction by macaque serum at 70 dpv (n = 3 macaques in Ctrl, Low, and Middle groups; n = 3 repeats in H-M group; n = 7 macaques in High group; n = 16 macaques in Day 0 group).

(G) Quantitative results of IC50 in e. Mean IC50 of H-M is represented by red bar. Positive cutoff of IC50 is represented by dotted line.

(H) ELISA of RBD antibodies from high-dose macaque serum in group 1 at 0, 42, 70, 126, 182, 273, 364 and 598 dpv (n = 3 macaques in each group; n = 3 repeats in H-M group).

(I) Competitive ELISA of inhibition of SARS-CoV-2 RBD-hACE2 interactions by high-dose macaque serum in group 1 at 0, 42, 70, 126, 182, 273, 364 and 598 dpv (n = 7 macaques in Day 0, 42, 70 group; n = 3 macaques in Day 126, 182, 273, 364, 598 group; n = 3 repeats in H-M group).

Values are means ± SEM or geometric mean + geometric standard deviation for antibody titer. *: *P* < 0.05; **: *P* < 0.01; ***: *P* < 0.001.

**
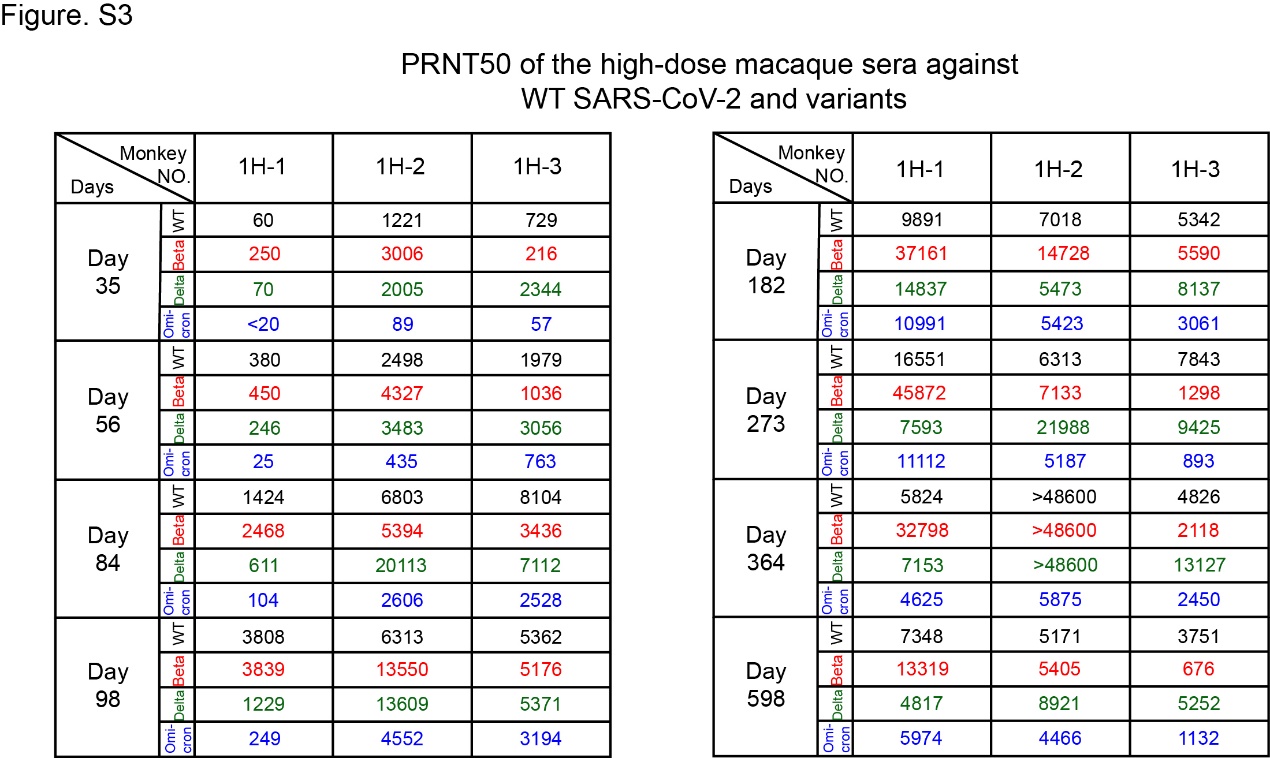
Figure S3. PRNT 50 values of high-dose macaque sera (group 1) from 35 to 598 dpv against wild-type SARS-CoV-2, Beta, Delta and Omicron variants.**

**
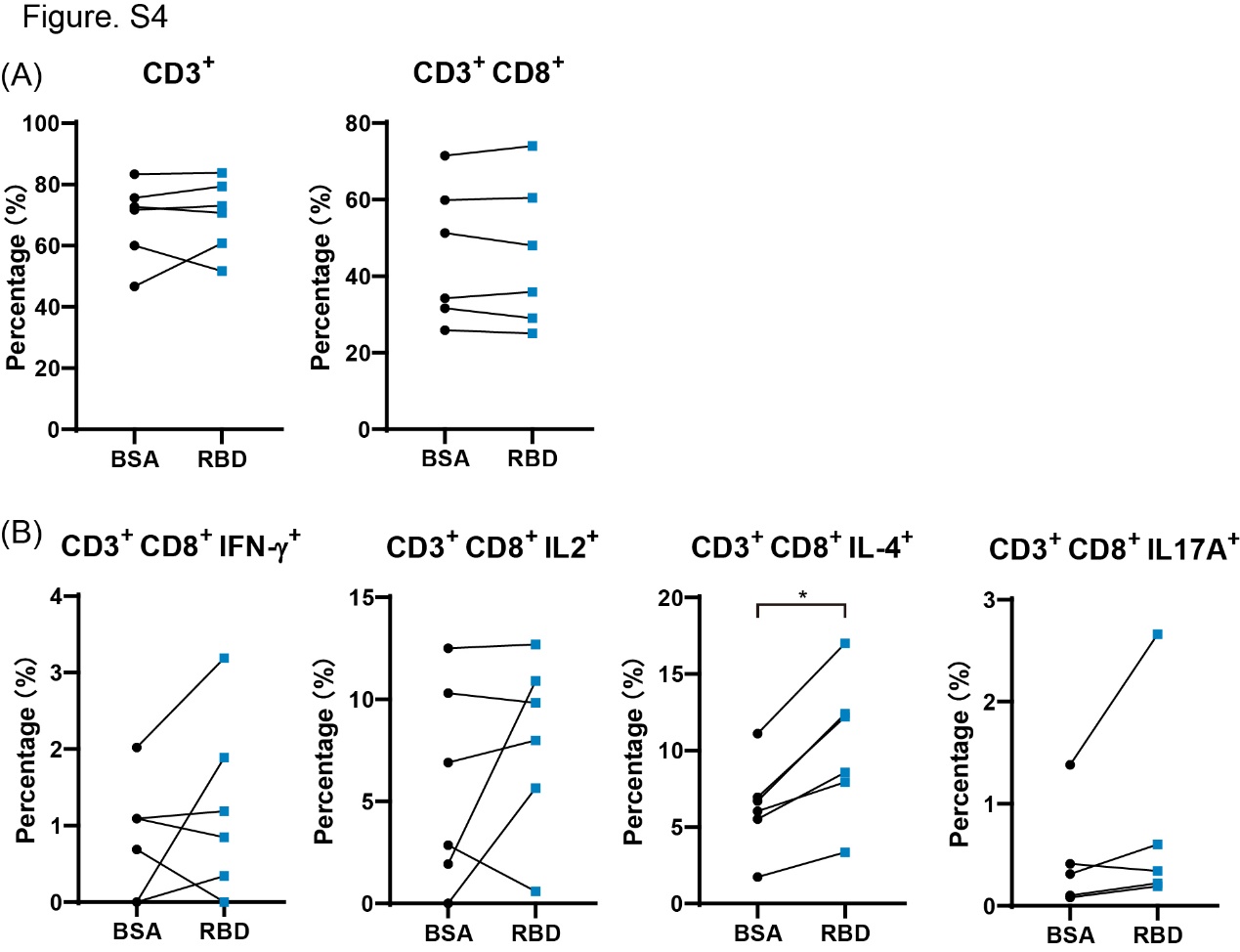
**

**Figure S4. Changes of immune cells after vaccination with AAV vaccine.**

(A) Percentage of CD3^+^and CD3^+^ CD8^+^ cells in blood of high-dose rhesus macaques at day 35 dpv activated by BSA or RBD peptide.

(B) Percentage of CD3^+^ CD8^+^ IFN-γ^+^, CD3^+^ CD8^+^ IL-2^+^, CD3^+^ CD8^+^ IL-4^+^ and CD3^+^ CD8^+^IL-17A^+^ cells in blood of high-dose rhesus macaques at day 35 dpv activated by BSA or RBD peptide.

n = 6 macaques in each group, Values are means ± SEM. *: *P* < 0.05.

**
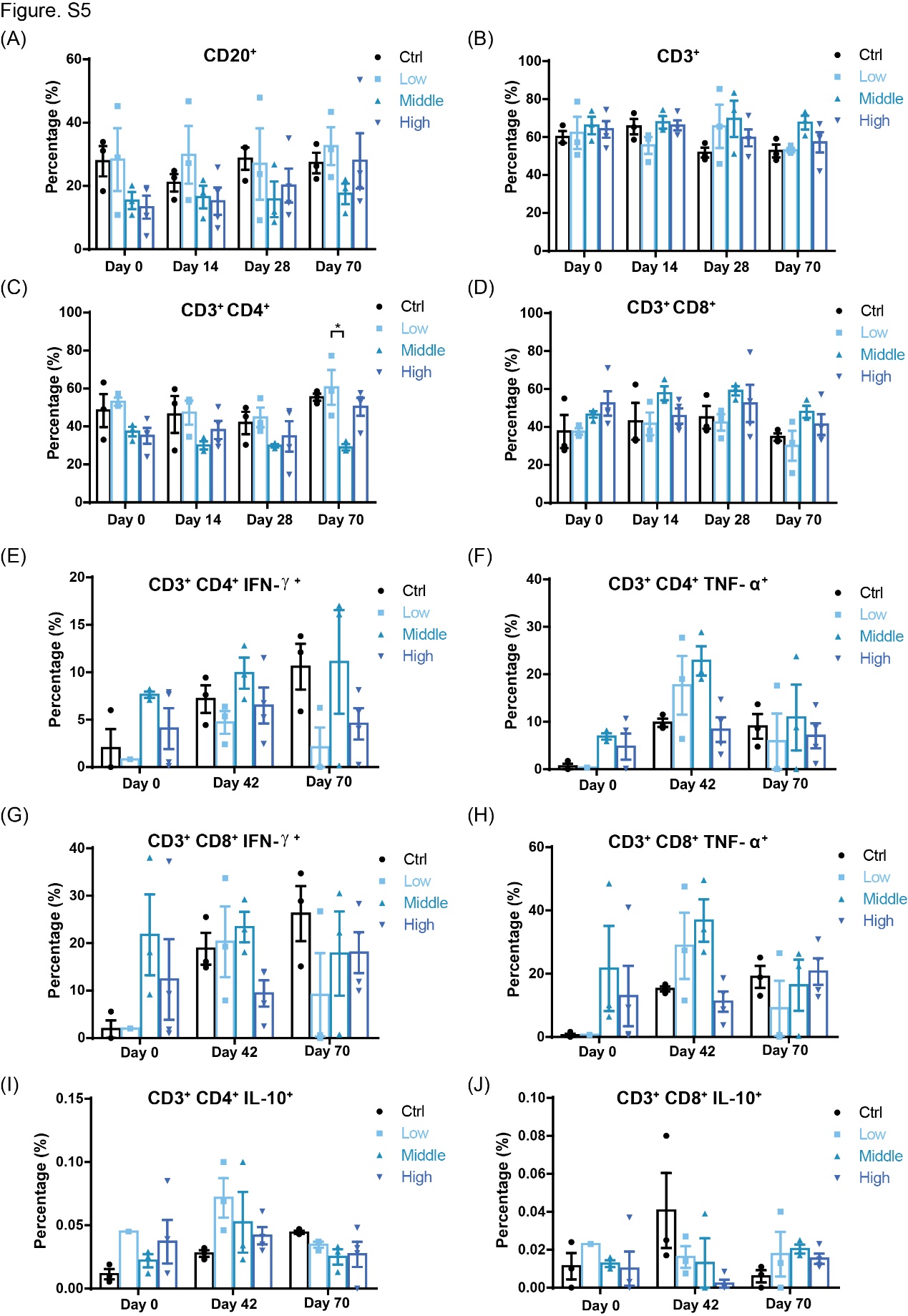
**

**Figure S5. Safety evaluation of SRBD vaccine with immune cells in rhesus macaques for 70 dpv.**

(A) Percentage of CD20^+^ cells in serum of high/middle/low-dose and control rhesus macaques at 0, 14, 28 and 70 dpv (Ctrl: intramuscular injection with high-dose AAV-CAG-GFP control; Low/Middle/High: intramuscular injection of low/middle/high-dose SRBD vaccine).

(B) Percentage of CD3^+^ cells in serum of high/middle/low-dose and control rhesus macaques at 0, 14, 28 and 70 dpv.

(C) Percentage of CD3^+^ and CD4^+^ cells in serum of high/middle/low-dose and control rhesus macaques at 0, 14, 28 and 70 dpv.

(D) Percentage of CD3^+^ and CD8^+^ cells in serum of high/middle/low-dose and control rhesus macaques at 0, 14, 28 and 70 dpv.

(E) Percentage of CD3^+^, CD4^+^, and IFN-γ^+^ cells in serum of high/middle/low-dose and control rhesus macaques at 0, 42 and 70 dpv.

(F) Percentage of CD3^+^, CD4^+^, and TNF-α^+^ cells in serum of high/middle/low-dose and control rhesus macaques at 0, 42 and 70 dpv.

(G) Percentage of CD3^+^, CD8^+^, and IFN-γ^+^ cells in serum of high/middle/low-dose and control rhesus macaques at 0, 42 and 70 dpv.

(H) Percentage of CD3^+^, CD8^+^, and TNF-α^+^ cells in serum of high/middle/low-dose and control rhesus macaques at 0, 42 and 70 dpv.

(I) Percentage of CD3^+^, CD4^+^, and IL-10^+^ cells in serum of high/middle/low-dose and control rhesus macaques at 0, 42 and 70 dpv.

(J) Percentage of CD3^+^, CD8^+^, and IL-10^+^ cells in serum of high/middle/low-dose and control rhesus macaques at s 0, 42 and 70 dpv.

n = 3 macaques in Ctrl, Low, and Middle groups; n = 4 macaques in High group, Values are means ± SEM. *: *P* < 0.05.

**
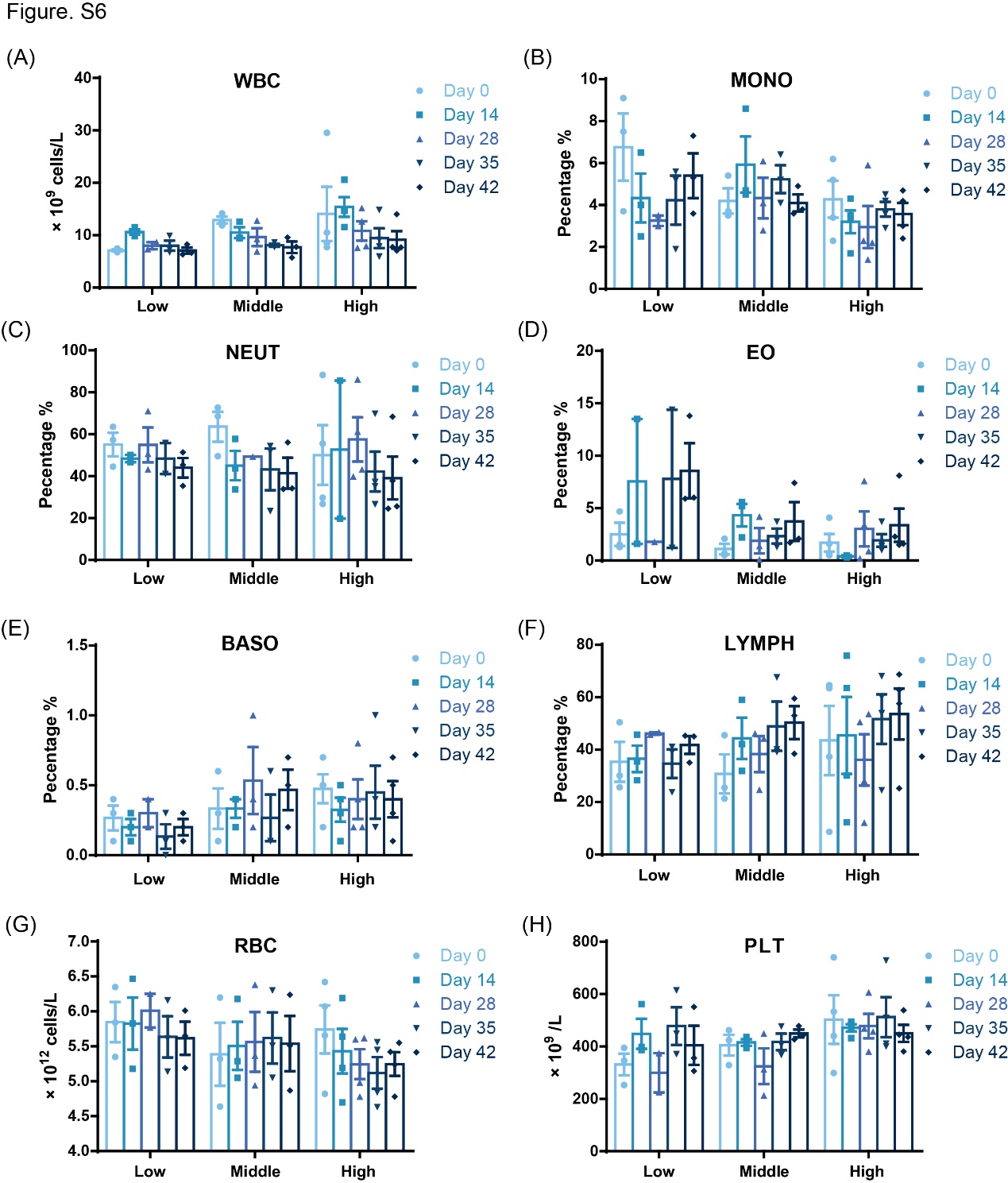
Figure S6. Pathological indicators in blood of macaques following intramuscular injection of AAV-SRBD vaccine.**

(A) White blood cell (WBC) count in serum of high/middle/low-dose rhesus macaques at 0, 14, 28, 35, and 42 dpv.

(B) Monocyte cell (MONO) percentage in serum of high/middle/low-dose rhesus macaques at 0, 14, 28, 35, and 42 dpv.

(C) Neutrophil cell (NEUT) percentage in serum of high/middle/low-dose rhesus macaques at 0, 14, 28, 35, and 42 dpv.

(D) Eosinophil cell (EO) percentage in serum of high/middle/low-dose rhesus macaques at 0, 14, 28, 35, and 42 dpv.

(E) Basophil cell (BASO) percentage in serum of high/middle/low-dose rhesus macaques at 0, 14, 28, 35, and 42 dpv.

(F) Lymphocyte cell (LYMPH) percentage in serum of high/middle/low-dose rhesus macaques at 0, 14, 28, 35, and 42 dpv.

(G) Red blood cell (RBC) count in serum of high/middle/low-dose rhesus macaques at 0, 14, 28, 35, and 42 dpv.

(H) Platelet (PLT) count in serum of high/middle/low-dose rhesus macaques at 0, 14, 28, 35, and 42 dpv.

n = 3 macaques in Low and Middle groups; n = 4 macaques in High group, Values are means ± SEM

**
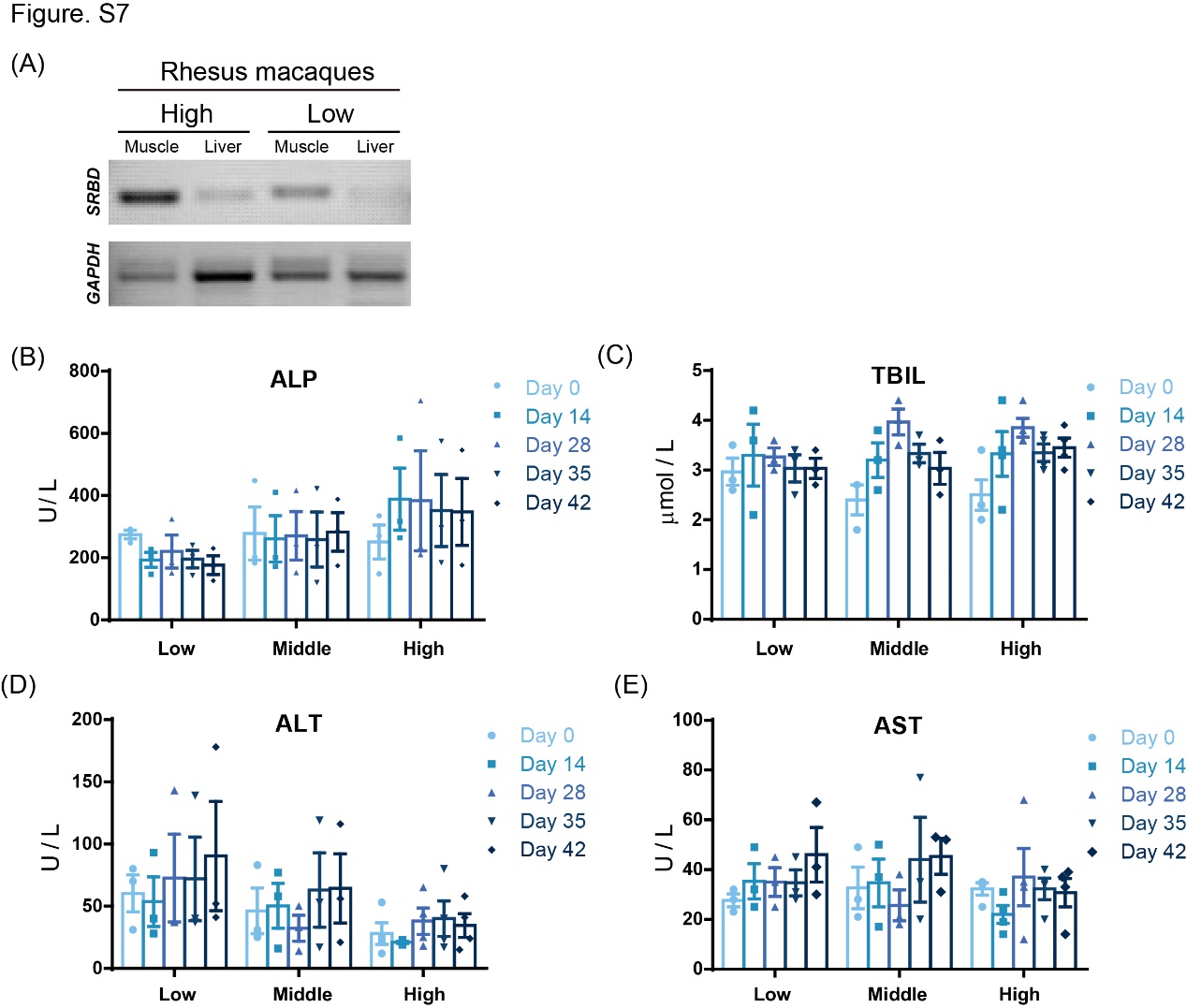
Figure S7. Infection of AAV in muscles and liver of macaques and hepatic function of the immunized macaques.**

(A) RT-PCR of RBD in muscle and liver cells from high- and low-dose macaques (70 dpv).

(B) Alkaline phosphatase (ALP) level in serum of high/middle/low-dose rhesus macaques at 0, 14, 28, 35, and 42 dpv.

(C) Total bilirubin (TBIL) level in serum of high/middle/low-dose rhesus macaques at 0, 14, 28, 35, and 42 dpv.

(D) Alanine aminotransferase (ALT) level in serum of high/middle/low-dose rhesus macaques at 0, 14, 28, 35, and 42 dpv.

(E) Aspartate aminotransferase (AST) level in serum of high/middle/low-dose rhesus macaques at 0, 14, 28, 35, and 42 dpv.

n = 3 macaques in Low and Middle groups; n = 4 macaques in High group, Values are means ± SEM

**
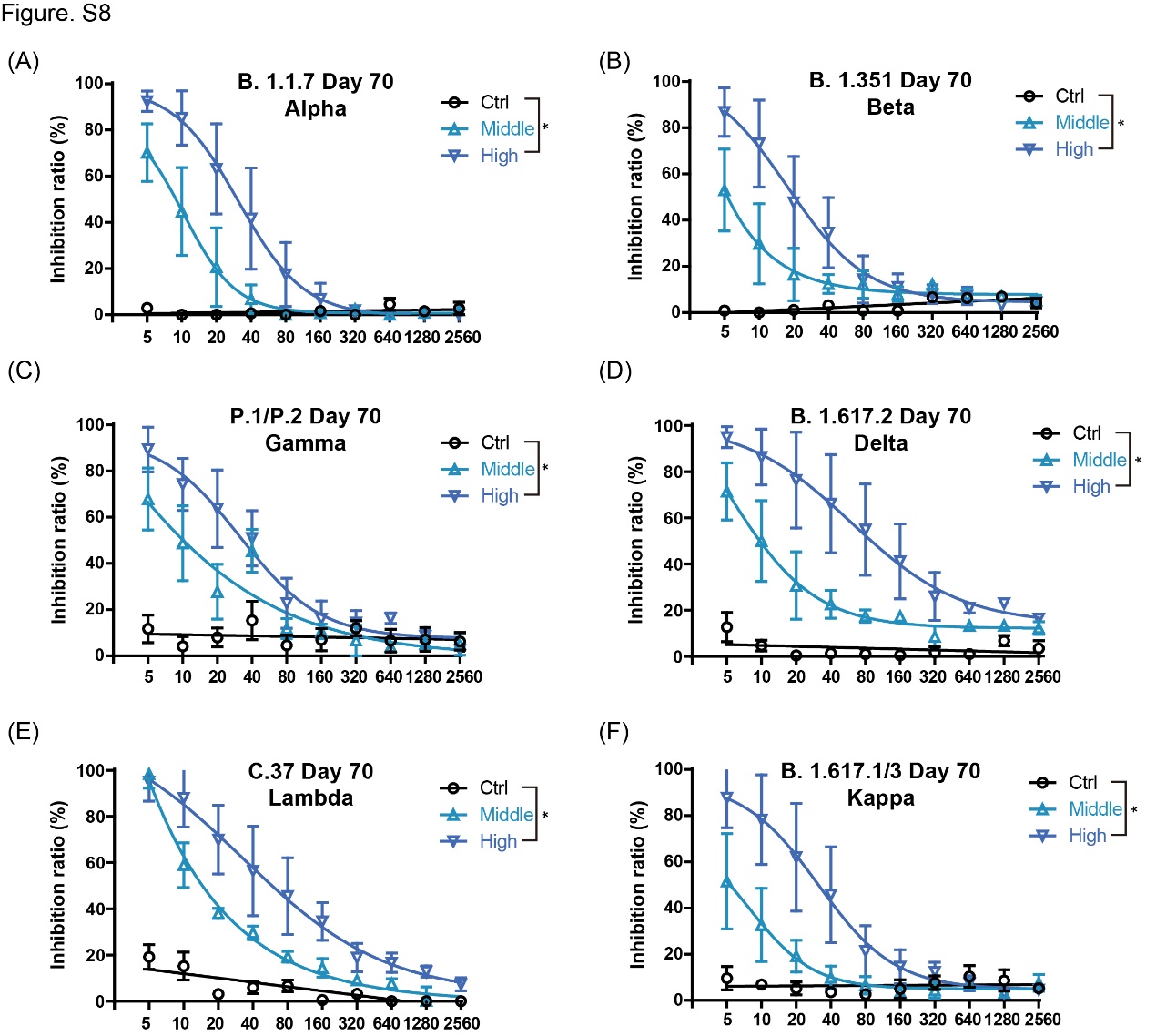
Figure S8. AAV-SRBD immune serum efficiently inhibited the binding of ACE2 with RBD variants.**

(A) Competitive ELISA of inhibition of RBD domain in Alpha variant and hACE2 interactions by macaque serum at 70 dpv. (Middle/High: 70 dpv of middle/high-dose SRBD vaccine; Ctrl: 70 dpv of high-dose AAV-CAG-GFP control; n = 3 macaques in each group).

(B) Competitive ELISA of inhibition of RBD domain in Beta variant and hACE2 interactions by macaque serum at 70 dpv (n = 3 macaques in each group).

(C) Competitive ELISA of inhibition of RBD domain in Gamma variant and hACE2 interactions by macaque serum at 70 dpv (n = 3 macaques in each group).

(D) Competitive ELISA of inhibition of RBD domain in Delta variant and hACE2 interactions by macaque serum at 70 dpv (n = 3 macaques in each group).

(E) Competitive ELISA of inhibition of RBD domain in Lambda and hACE2 interactions by macaque serum at 70 dpv (n = 3 macaques in each group).

(F) Competitive ELISA of inhibition of RBD domain in Kappa variant and hACE2 interactions by macaque serum at 70 dpv (n = 3 macaques in each group).

Values are means ± SEM. *: *P* < 0.05.
